# Recombinant Adeno-Associated Virus Serotype 6 Efficiently Transduces Primary Human Melanocytes

**DOI:** 10.1371/journal.pone.0062753

**Published:** 2013-04-30

**Authors:** Hilary M. Sheppard, James E. Ussher, Daniel Verdon, Jennifer Chen, John A. Taylor, P. Rod Dunbar

**Affiliations:** 1 School of Biological Sciences, University of Auckland, Auckland, New Zealand; 2 Maurice Wilkins Centre for Molecular Biodiscovery, University of Auckland, Auckland, New Zealand; University of Colorado, United States of America

## Abstract

The study of melanocyte biology is important to understand their role in health and disease. However, current methods of gene transfer into melanocytes are limited by safety or efficacy. Recombinant adeno-associated virus (rAAV) has been extensively investigated as a gene therapy vector, is safe and is associated with persistent transgene expression without genome integration. There are twelve serotypes and many capsid variants of rAAV. However, a comparative study to determine which rAAV is most efficient at transducing primary human melanocytes has not been conducted. We therefore sought to determine the optimum rAAV variant for use in the *in vitro* transduction of primary human melanocytes, which could also be informative to future *in vivo* studies. We have screened eight variants of rAAV for their ability to transduce primary human melanocytes and identified rAAV6 as the optimal serotype, transducing 7–78% of cells. No increase in transduction was seen with rAAV6 tyrosine capsid mutants. The number of cells expressing the transgene peaked at 6–12 days post-infection, and transduced cells were still detectable at day 28. Therefore rAAV6 should be considered as a non-integrating vector for the transduction of primary human melanocytes.

## Introduction

Melanocytes are found in the skin, iris, and uvea and are responsible for the pigmentation which protects against the mutagenesis induced by ultraviolet radiation. Melanocyte dysfunction is important in the pathology of several heritable diseases, including albinism, as well as numerous disorders of pigmentation with more complex causes, such as vitiligo. Studies of melanocyte function are also highly relevant to the study of their malignant transformation, especially now that the first molecularly-targeted drugs with clinical efficacy against malignant melanoma are becoming available [1]. Vectors that efficiently transduce human melanocytes are therefore relevant to both fundamental studies of melanocyte biology and translational research.

While transduction of primary human melanocytes by gamma-retroviral vectors has been reported [2], [3], [4], geneticin selection or co-culture with infected feeder cells, keratinocytes, or producer cell lines have been required to achieve high levels of transduction. Furthermore, retroviruses are unable to transduce non-dividing cells [5] and integration of the vector genome may lead to malignant transformation [6]. Lentiviral vectors have shown more promise, efficiently transducing primary human melanocytes with transduction rates higher than 90% and with transgene expression maintained for at least 4 weeks [7]. Lentiviral vectors have been widely used in *in vitro* studies of melanocyte biology 8,9,10, however their clinical use may be limited by the risk of insertional mutagenesis, although this risk may be lower than with gamma-retroviral vectors [11], [12]. While primary melanocytes can be transduced by adenoviral vectors [13], [14] their utility is limited by a lack of persistence and the immunogenicity of the vector [15], [16].

Non-viral methods of gene transfer to melanocytes have also been reported. While the efficiency of transduction with lipid-based transfection reagents is low (<5%) [17], [18], high rates of transduction of primary human melanocytes have been achieved with the Nucleofector™ (44%), although viability was significantly affected [18].

Adeno-associated virus (AAV) is a helper-dependent parvovirus that is not associated with any known pathology. Recombinant AAV (rAAV) expresses no viral proteins, is able to transduce both dividing and non-dividing cells, and is associated with persistent transgene expression [19], [20]. It has been extensively evaluated as a gene therapy vector with hundreds of patients having received rAAV in clinical trials without any significant vector-associated complications [21], [22], [23], [24], [25], [26]. Unlike gamma-retroviral and lentiviral vectors, integration of rAAV is uncommon, with the vector genome persisting in an episomal state [27]. There are twelve serotypes and many capsid variants of rAAV, with different receptor and co-receptor usage resulting in differing tropisms for different cell types [28], [29], [30]. While no transduction of primary human melanocytes was seen with a serotype 2 rAAV vector (rAAV2) in a previous study [31], other serotypes have not been assessed in human melanocytes. Transduction of murine choroid and iris melanocytes by rAAV serotype 1 following intraocular injection has been reported [32], suggesting at least some rAAV serotypes might be capable of transducing human melanocytes. We therefore sought to determine the ability of eight different capsid variants of rAAV to transduce primary human melanocytes.

## Materials and Methods

### Culture of Primary Melanocytes and Melanoma Cell Lines

Primary human melanocytes (from five donors) were purchased from Invitrogen (Cat. No. C-024-5C for lightly pigmented cells and Cat. No. C-024-5C for medium pigmented cells) and grown in Cascade medium (Invitrogen, Cat. No. M-254-500) supplemented with Cascade Human Melanocyte Growth Supplement (Invitrogen, Cat. No. S-002-5) (hereafter referred to as complete media). Melanoma cell lines (SK-MEL-23 [33], SK-MEL-29 [34], and Trombelli [35] (a kind gift from Prof. Vincenzo Cerundolo, University of Oxford) were grown in RPMI 1640 (Invitrogen, Cat. No. 22400-089) supplemented with 10% foetal bovine serum (FBS) (Invitrogen, Cat. No. 10099-141), 1X penicillin-streptomycin (Invitrogen, Cat. No. 15140) and 1X GlutaMAX-1 (PSG) (Invitrogen, Cat. No. 35050). Cells were incubated at 37°C/5% CO2.

### Production of rAAV

**Figure 1:**
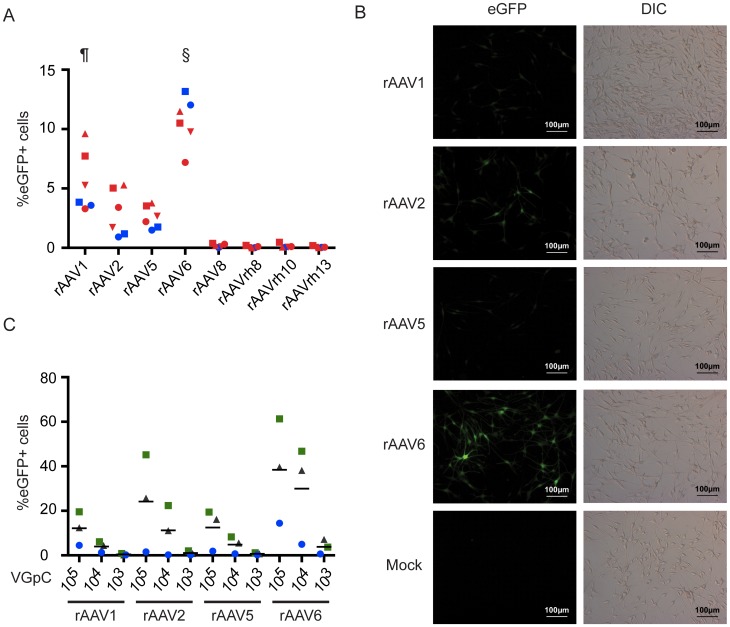
Comparison of the efficiency of transduction of primary human melanocytes by rAAV variants. (A) The percentage of primary human melanocytes expressing eGFP 48 hours post infection with the indicated rAAV/eGFP variants (10^5^ vector genomes per cell) as assessed by flow cytometry. Cells from two donors at various passages were used (n = 6). ^§^AAV6 versus all others, p<0.05, ^¶^AAV1 versus all others except AAV6, p<0.05. (B) Representative fluorescent microscopy images of primary human melanocytes 48 hours post infection with the indicated rAAV variants. (C) The effect of vector genome copies per cell (VGpC) on the percentage of primary human melanocytes expressing eGFP 48 hours post infection as assessed by flow cytometry. Cells from three donors were used, including one also used in (A). The data from (C) is re-presented in Figure S1 as normalised to the maximal level of transduction seen in each donor. Data points represent the average of triplicate repeats. Different colours represent different donors and are consistent throughout the paper. The various symbols refer to cells of different passage number from those donors.

Recombinant AAV was produced as previously described [36], [37]. Briefly, HEK293 T cells were triple transfected with a plasmid encoding the vector genome (pAM/CAG-eGFP-WPRE-bGHpA), a packaging plasmid (pH21, pNLrep, pH25A, pAAV2/6, pBR18-D2/8, pAAV2/rh8c, pAAV2/rh10, or pAAV2/rh13R), and the helper plasmid, pFΔ6. Serotype 6 capsid mutants were produced using the previously described packaging plasmids pAAV2/6(Y445F), pAAV2/6(Y731F), and pAAV2/6(Y445F+Y731F) [36]. Plasmids pAM/CAG-eGFP-WPRE-bGHpA, pH21, pNLrep, pH25A, pBR18-D2/8, and pFΔ6 were kindly provided by Dr Deborah Young (University of Auckland), and plasmids pAAV2/6, pAAV2/rh8c, pAAV2/rh10, and pAAV2/rh13R were kindly provided by Professor James Wilson (University of Pennsylvania). Forty eight hours post transfection cells were harvested and the vector purified by ultracentrifugation on a discontinuous iodixanol gradient, followed by anion exchange chromatography (all serotypes) or heparin affinity chromatography (rAAV2 and rAAV6) as previously described [36]. Alternatively rAAV6 was purified by heparin affinity chromatography alone [37]. All the vectors used in figure 1 were purified by ultracentrifugation followed by chromatography. In the other figures, rAAV2 and rAAV6 vectors used in the same experiment were purified by the same method. Vectors were titred by quantitative real time PCR. All variants were infectious in a transduction assay in HeLa cells [36].

### Transduction of Melanocytes and Melanoma Cell Lines

Cells were seeded at 2×10^4^ cells per well of a 96 well plate 1 day prior to infection in 200 µL of media. On the day of infection 150 µL of media was removed, the vector added at the indicated number of vector genomes per cell and the cells incubated at 37°C/5% CO2 for 3 hours. The media was then replaced with 200 µL of fresh media and the cells incubated for a further 48 hours unless otherwise indicated.

### Flow Cytometry

Cells were removed from plates by incubation with trypLE (Invitrogen), washed, resuspended in FACS buffer (PBS +1% foetal bovine serum), and analysed on a FACS Calibur (BD Biosciences) with CellQuest software (BD Biosciences) or a FACS Aria (BD Biosciences with DIVA software (BD Biosciences).

### Fluorescent Microscopy

Live transduced cells were washed with TBS buffer to remove residual culture medium, covered with a coverslip and then visualized with a Leica DMRE Fluorescent microscope equipped with the epi-fluorescent filter 470–490 µm (Leica Microsystems, Heerbrugg, Switzerland). Differential interference contrast (DIC) and fluorescent images were acquired at room temperature using 20×/0.50 NA Leica objectives, a Leica DC500 Digital camera and analySIS FIVE software (Olympus, Tokyo, Japan). Images were processed and figures were generated using Cytosketch image analysis software (CytoCode, Auckland, New Zealand, www.cytocode.com).

### Quantification of Internalised Virus by Real Time PCR

Seven or 24 days post transduction cells were washed in PBS, removed from the plates by incubation with trypLE (Invitrogen), washed and counted. 100,000 cells were then pelleted and resuspended in 50 µL prepGEM Tissue reagent (ZyGEM, cat. no. PT10050) and incubated for 15 minutes at 75°C, then 10 minutes at 95°C. 0.2 µL of this extract was used directly in a quantitative real time PCR, as described previously [36]. The number of vector genomes per sample was calculated from a dilution series of linearised plasmid. Samples were normalised to the amount of GAPDH DNA.

### Statistical Analysis

Individual data points are shown. Multiple groups were compared using a one-way ANOVA with Tukey’s or Sidak’s multiple comparison test as appropriate using Prism 6.0 (GraphPad Software, Inc).

## Results

Eight variants of rAAV were assessed for their ability to transduce primary melanocyte cell lines. Cell lines from two donors were transduced at several different passages with the rAAV variants at 10^5^ vector genomes per cell. Transduction was assessed by detection of the vector-encoded eGFP transgene by flow cytometry. At 48 hours post infection rAAV1, rAAV2, rAAV5, and rAAV6 consistently transduced more than 1% of cells, while no transduction was seen with rAAV8, rAAVrh8, rAAVrh10, and rAAVrh13 (Figure 1A). The highest levels of transduction were consistently seen with rAAV6, with a mean of 10.7% of cells transduced. Consistent with transgene expression, few vector genomes were detected by quantitative real time PCR in melanocytes infected with rAAVrh10; in contrast the number of vector genomes per cell was more than 1000-fold higher following infection with rAAV5 or rAAV6. Transduction of melanocytes by rAAV1, rAAV2, rAAV5, and rAAV6 was confirmed by fluorescent microscopy with the highest rates of transduction and levels of transgene expression seen with rAAV6 (Figure 1B).

The effect of the number of vector genomes per cell on transduction efficiency was assessed with rAAV1, rAAV2, rAAV5, and rAAV6 in three cell lines, including two from previously untested donors (Figure 1C, Figure S1). Again, rAAV6 was the most efficient serotype, with up to 61% of cells transduced with 10^5^ vector genomes per cell. With 10^4^ vector genomes per cell rAAV6 consistently transduced a greater percentage of melanocytes than the other serotypes at a 10-fold higher dose, although this did not reach statistical significance.

**Figure 2:**
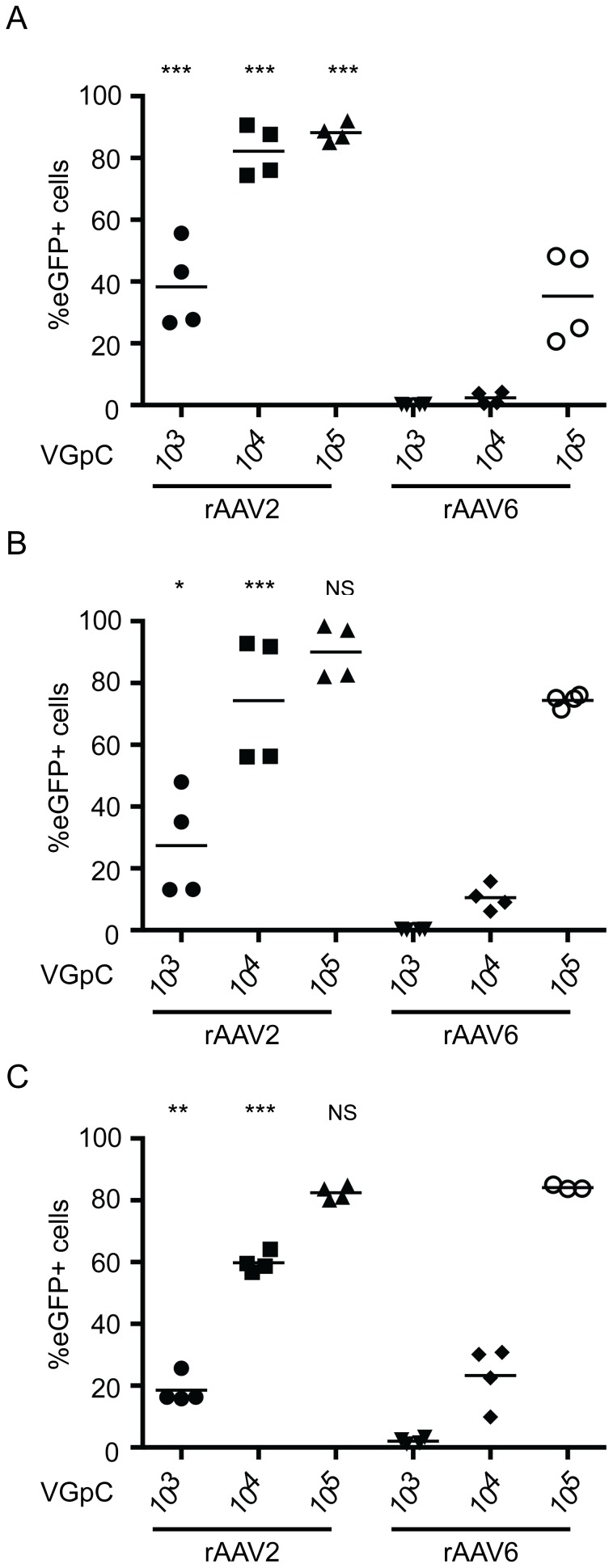
Melanoma cell lines are efficiently transduced by rAAV. The percentage of (A) Trombelli, (B) SkMel23 and (C) SkMel29 expressing eGFP 48 hours post infection with increasing concentrations of either rAAV2 or rAAV6 as assessed by flow cytometry (VGpC = vector genome copies per cell). All comparisons are between rAAV2 and rAAV6 at the same concentration. *p<0.05;**p<0.01; ***p<0.001; NS not significant. Data points represent the average of duplicate repeats.

Next we assessed whether rAAV6 was also better than rAAV2 at transducing melanoma cell lines. Three melanoma cell lines, Trombelli, SK-MEL-23, and SK-MEL-29, were transduced with rAAV6 and AAV2 at a range of concentrations. In contrast to primary human melanocyte cell lines all three melanoma cell lines were more efficiently transduced by rAAV2 (Figure 2). Furthermore, even at low virus concentrations high rates of transduction were seen with rAAV2.

**Figure 3:**
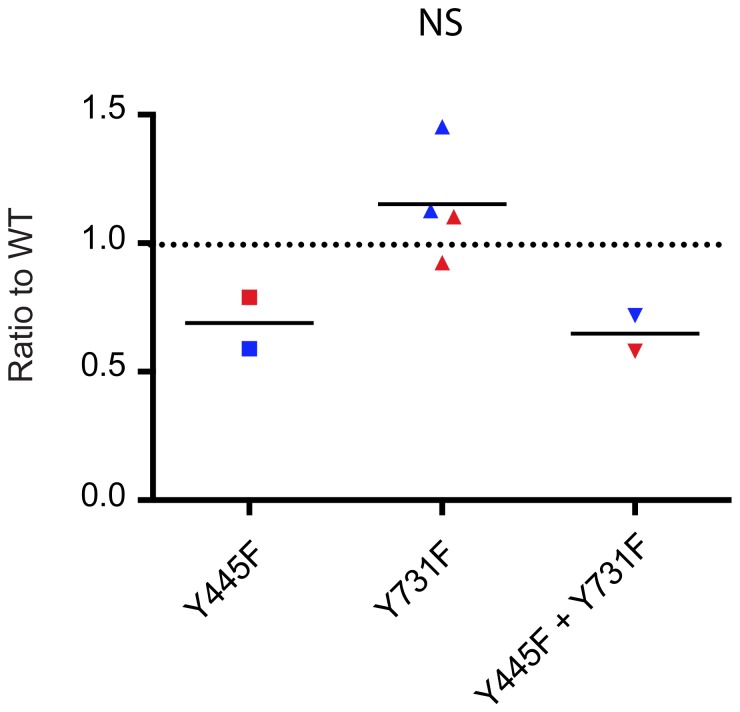
Mutation of surface-exposed tyrosine residues on the rAAV6 capsid does not increase transduction of primary human melanocytes. Melanocytes were transduced with wild type rAAV6, rAAV6 Y444F, rAAV6 Y731F, or the double mutant rAAV6 Y444F+Y731F at 10^5^ vector genomes per cell and eGFP expression assessed by flow cytometry 48 hours post infection. Data points represent the average of technical replicates (n = 1−3) in two donors. Results are expressed relative to wild type.

A rate limiting step in the transduction of certain cell lines by rAAV2 is the proteosomal degradation of vector capsids secondary to phosphorylation, and subsequent polyubiquitination, of surface exposed tyrosine residues by epidermal growth factor receptor protein tyrosine kinase [38]. Mutation of surface exposed tyrosine residues in the capsid to phenylalanine residues increases transduction of certain cell lines by both rAAV2 and rAAV6 [36], [37], [38]. Therefore we assessed the ability of three previously reported rAAV6 capsid mutants, which more efficiently transduce HeLa cells [36], to transduce primary human melanocyte cell lines (Figure 3). A small increase in transduction efficiency compared with wild type was seen with the Y731F mutant, although this did not reach significance. In contrast, the Y445F mutant and the double mutant were associated with a reduction in transduction efficiency, as previously seen with monocyte-derived dendritic cells and human adipose-derived stem cells [36], [37].

**Figure 4:**
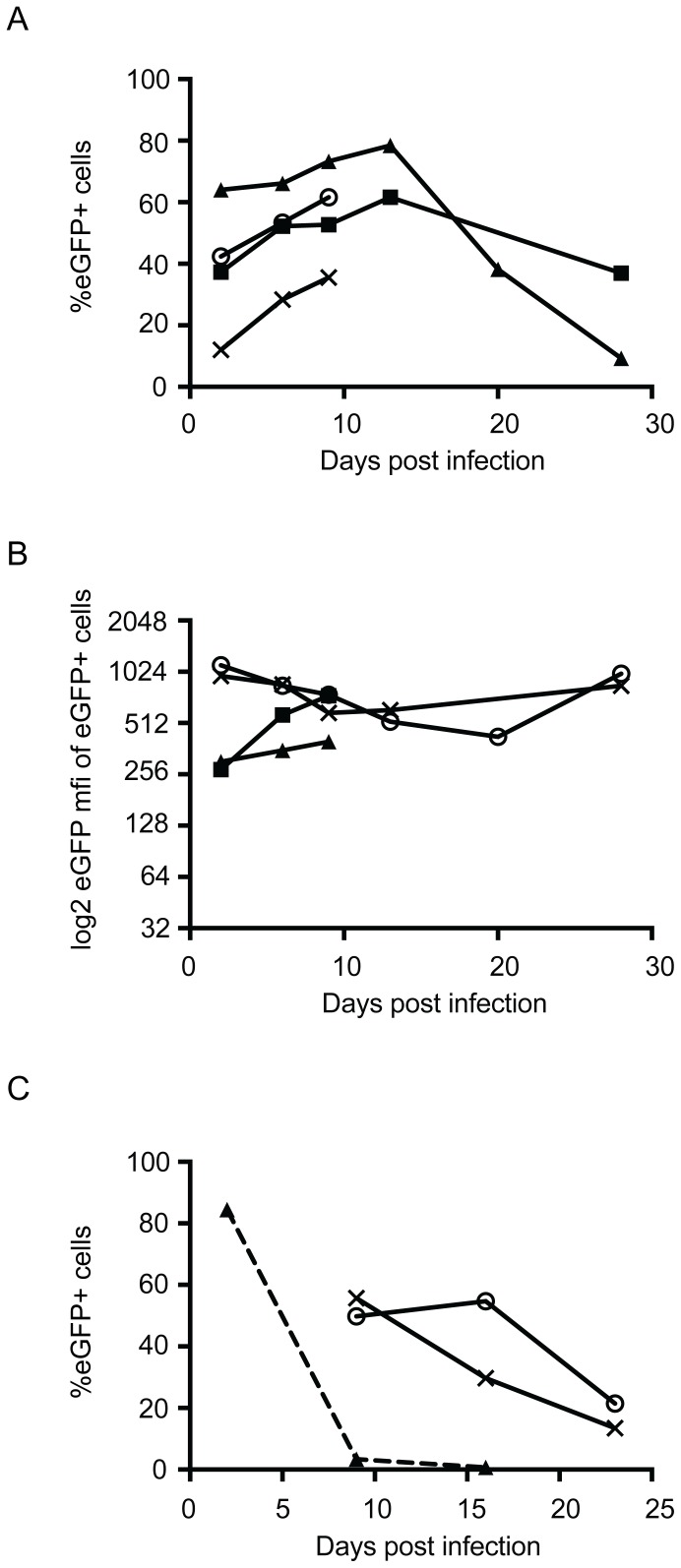
Timecourse of transgene expression by primary human melanocytes following transduction with rAAV6. (A) Variation with time in the percentage of primary human melanocytes expressing eGFP and (B) the levels of transgene expression in eGFP positive cells as assessed by mean fluorescent intensity (mfi) of positive cells. Cells were infected with 10^5^ vector genomes per cell of rAAV6 and eGFP expression assessed by flow cytometry at days 2, 6, 9 (all four donors), 13, 20, and 28 (two donors). Different symbols represent different donors. Data points represent the average of duplicate repeats. (C) The effect of cell division on the loss of eGFP expression. One rapidly dividing melanoma cell line (SKMel23, represented by the triangle symbol) and 2 slower dividing primary melanocyte cultures (represented by a cross [medium pigmented] or open circle [lightly pigmented]) were infected with 10^5^ vector genomes per cell of rAAV6 and eGFP expression assessed by flow cytometry at either days 2, 9 and 16 (SKMel23) or days 9, 16 and 23 (primary melanocytes). Data points represent the average of triplicate samples.

To assess how transgene expression changed with time, melanocytes were infected with rAAV6 and transduction assessed at days 2, 6, and 9 post infection; with two cell lines transduction was assessed out to 28 days. The maximal percentage of cells expressing the transgene was seen at days 9–12 (Figure 4A). While transduced melanocytes were still detectable 28 days post-infection, the percentage of cells expressing the transgene was less than at 48 hours post-infection (mean 39% at 48 hours vs 23% at day 28). However, the level of expression of the transgene in eGFP-positive cells, as assessed by mean fluorescent intensity, remained relatively constant over the time course (Figure 4B). The loss of eGFP-expressing cells observed at later time points correlated with cell division (Figure 4C). While transgene-expressing cells were quickly lost from cultures of the rapidly growing melanoma cell line, SK-MEL23 (passaged ∼1∶12 weekly), they were lost much more slowly from melanocyte cultures, with the rate of loss slower in the lightly pigmented cell line (passaged weekly ∼1∶2) than the medium pigmented cell line (passaged weekly ∼1∶3).

## Discussion

We screened eight capsid variants of rAAV and identified rAAV6 as the optimal serotype for the transduction of primary human melanocytes. Between 7% and 78% of cells were transduced by rAAV6 at 10^5^ vector genomes per cell while lower rates of transduction were seen with rAAV1 (3–20%), rAAV2 (0.7–46%), and rAAV5 (1.5–19%). Even at a 10-fold lower concentration, rAAV6 virus consistently transduced more melanocytes than rAAV1, rAAV2, and rAAV5 at the highest concentration of 10^5^ vector genome copies per cell. We also observed that AAV6 yielded the highest levels of transgene expression compared to the other serotypes (Figure 1C and data not shown).

Only one previous study has investigated the transduction of primary human melanocytes by rAAV with no transduction seen with rAAV2 [31]. Melanocytes were infected at a similar vector concentration (equivalent to our 10^5^ viral genome copies per cell) but had been cultured for a shorter time *ex vivo* (between 2–7 days) than in our experiments. While we noted no association between passage number and transduction efficiency we cannot exclude the possibility that freshly isolated cells are more resistant to transduction. Successful transduction of mouse choroid and iris melanocytes has been reported following intraocular injection of rAAV1 [32]. Interestingly, retinal pigment epithelium, the other pigmented cell type in the eye, can also be transduced by rAAV but rAAV6 is less efficient than other serotypes [39], [40], [41], [42], highlighting the need to determine the optimal serotype for each cell type experimentally.

In contrast to primary human melanocytes, the transduction of melanoma cell lines by rAAV has previously been reported. Li *et al* developed a chimeric rAAV by DNA shuffling that had increased ability to transduce a range of human and mouse melanoma cell lines compared with the parental serotypes (1, 2, 8, and 9), however it failed to transduce two melanoma cell lines that had been in culture for less than 14 days [43]. Indeed, we found three melanoma cell lines (Trombelli, SKMel23, and SKMel29) to be readily transduced by both rAAV2 and rAAV6, although higher rates of transduction were seen with rAAV2 than with rAAV6. The increased transducability of melanoma cells may reflect changes in the expression of surface, cytoplasmic, or nuclear proteins involved in rAAV transduction. Indeed, increased expression of heparan sulphate and fibroblast growth factor receptor 1, the receptor and co-receptor for AAV2 [44], [45], has been demonstrated in several melanoma cell lines [46], supporting the observation of increased transduction of melanoma cell lines by rAAV2. EGFR, a receptor for AAV6 [47], is expressed by both primary human melanocytes and melanoma cell lines [48]. However, we note that gene expression profiles can change when cells from melanoma metastases are cultured *in vitro* [49]. Therefore, whilst these studies are informative, further *in vivo* studies would be required to verify which is the optimum serotype to target melanoma or melanocyte cells *in vivo*.

Despite the high similarity of the AAV1 and AAV6 capsids (99.2%), rAAV6 transduced melanocytes approximately twice as efficiently as rAAV1. Both rAAV1 and rAAV6 utilise N-linked sialic acid on glycoproteins as their primary receptor [50]. As melanocytes express high levels of cell surface sialic acid when cultured *in vitro* [51], this may contribute to the efficient transduction seen with both these vectors. However, despite their high degree of similarity only rAAV6 has been to shown to bind heparin, which demonstrably affects its tissue tropism [52]. As melanocytes have been shown to express heparan sulfate proteoglycans (HPSG) [53] it is possible that this additional affinity mediates the higher levels of transduction observed with rAAV6 compared to rAAV1. HPSG is the primary receptor for AAV2 [54] and yet rAAV6 shows a three-fold increase in transduction efficiency over rAAV2 in our experiments. Again it may be the unique dual glycan receptor (heparan sulphate and sialic acid) usage of AAV6 [55] that mediates the superior tropism of rAAV6 for melanocytes above rAAV2.

Significant interdonor variation in the efficiency of transduction of primary human melanocytes was noted (compare figures 1A, 1C, and 4). We have previously noted significant interdonor variation in transduction of both human monocyte-derived dendritic cells and human adipose-derived stem cells [36], [37]. The reasons for this variation are unknown but may reflect differences in multiple steps in transduction, including receptor-mediated endocytosis, endosomal escape, nuclear trafficking, uncoating, and second strand synthesis.

Despite expression of EGFR by primary human melanocytes [48] little increase in transduction relative to wild type rAAV6 was seen with the tyrosine capsid mutant Y731F, while the Y445F mutant and the Y445F+Y731F double mutant were less efficient than wild type rAAV6. This is consistent with our previous findings in MoDCs and ASCs where a modest increase in transduction efficiency was seen with the Y731F mutant while there was a reduction in efficiency with the Y445F and Y445F+Y731F mutants [36], [37]. In contrast all mutants were more efficient than wild type rAAV6 at transducing HeLa cells [36] and increased transduction of mouse muscle with rAAV6 tyrosine capsid mutants has also been reported [56]. Therefore degradation of phosphorylated vector is not an important rate limiting step in the transduction of primary human melanocytes by rAAV6. Furthermore residue Y445 may be important in receptor binding, intracellular trafficking or uncoating of rAAV6 in melanocytes.

The number of cells transduced by rAAV6 continued to increase for 9–16 days post-infection (Figrues 4A and 4C) suggesting that steps in viral transduction other than viral entry may be rate limiting, such as vector uncoating or second strand DNA synthesis. Indeed, delayed vector uncoating has previously been shown to be responsible for the different rates of transduction of murine hepatocytes *in vivo* by serotypes 2, 6, and 8 [57]. Also, second strand DNA synthesis has been reported as the rate limiting step in transduction of various cell types and can be bypassed through the use of self-complementary vectors [58], [59]. The decrease in the percentage of cells expressing the transgene at later time points most likely reflects dilution with cell division of episomal vector (Figure 4C), however inactivation of the transgene or selective death of transduced cells cannot be excluded. Indeed we note that *in vitro* expression of eGFP over extended periods of time can be quite toxic to the cell [60]. Despite this loss of transgene expression a significant percentage of cells (mean of 23%) still expressed the transgene at day 28. It is important to note, however, that melanocyte cell division rates differ *in vivo* compared to *in vitro* [61], [62], [63] and therefore the kinetics of transgene expression demonstrated here are likely to differ from what would be observed *in vivo*. Indeed, more persistent transgene expression might be anticipated *in vivo* due to the lower rate of mitosis [61].

In summary, we have demonstrated that primary human melanocytes were efficiently transduced *in vitro* by rAAV1, rAAV2, rAAV5, and rAAV6, with the highest rates of transduction consistently seen with rAAV6. No further increase in transduction efficiency was seen by mutating surface-exposed tyrosine residues in the rAAV6 capsid. Transgene expression peaked 9 to 12 days post transduction and subsequently declined concomittant with cell division, although a significant percentage of cells still expressed the transgene 28 days post-tranduction. Therefore rAAV6 should be considered for future *in vitro* studies of human melanocyte biology and translational research.

## Supporting Information

Figure S1 **The effect of vector genome copies per cell (VGpC) on the percentage of primary human melanocytes expressing eGFP 48 hours post infection.** The data from Figure 1C is re-presented as normalised to the maximal level of transduction seen in each donor; in all cases this was with AAV6 at 10^5^ VGpC.
(TIF)
