# Supplementary figures and images for: Recombinant Adeno-Associated Virus Serotype 6 Efficiently Transduces Primary Human Melanocytes
Source: PLoS One. 2013 Apr 30;8(4):e62753. doi: 10.1371/journal.pone.0062753 (PMC3640030; doi:10.1371/journal.pone.0062753)

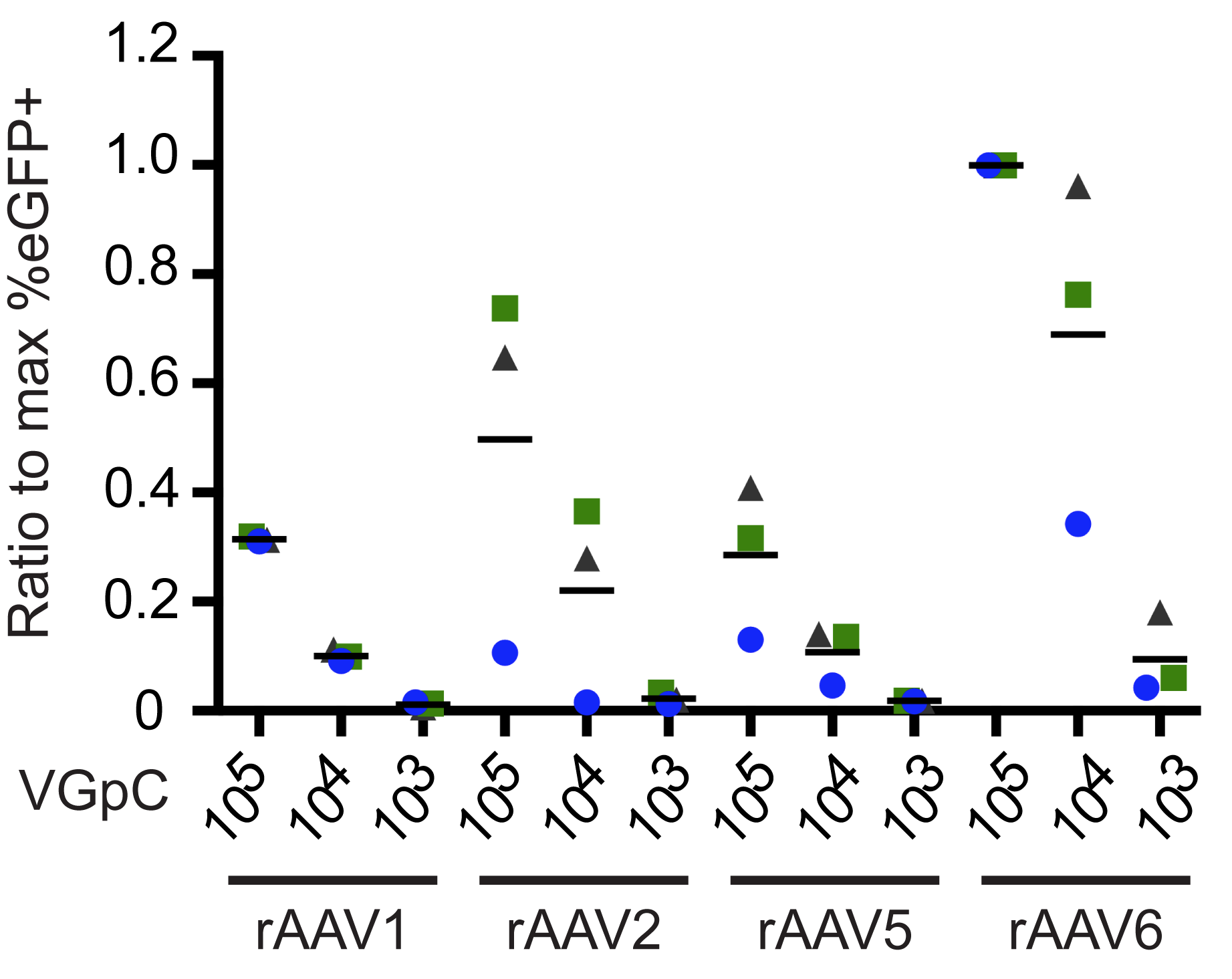

Supplement: Figure S1 — The effect of vector genome copies per cell (VGpC) on the percentage of primary human melanocytes expressing eGFP 48 hours post infection. The data from Figure 1C is re-presented as normalised to the maximal level of transduction seen in each donor; in all cases this was with AAV6 at 105 VGpC. (TIF) [file pone.0062753.s001.tif]
